# Intravitreal Dexamethasone Implant in the Treatment of Non-infectious Uveitis

**DOI:** 10.4274/tjo.galenos.2019.81594

**Published:** 2019-10-24

**Authors:** Murat Hasanreisoğlu, Hüseyin Baran Özdemir, Kaan Özkan, Murat Yüksel, Zeynep Aktaş, Hatice Tuba Atalay, Şengül Özdek, Gökhan Gürelik

**Affiliations:** 1 Gazi University Faculty of Medicine, Department of Ophthalmology, Ankara, Turkey; 2 University of Health Sciences, Ulucanlar Eye Training and Research Hospital, Ophthalmology Clinic, Ankara, Turkey

**Keywords:** Dexamethasone implant, uveitis, Ozurdex, intravitreal injection

## Abstract

**Objectives:**

To evaluate the long-term results of intravitreal dexamethasone implant (DEX) for noninfectious uveitis.

**Materials and Methods:**

The study included 62 eyes of 44 patients treated with DEX implant due to noninfectious uveitis and followed up for at least a year. Best-corrected visual acuity (BCVA), central foveal thickness, intraocular pressure (IOP), vitreous haze score, indications, immunomodulatory therapy and steroid usage before/after injection, number of injections, and adverse events were analyzed retrospectively.

**Results:**

Average follow-up was 20 months (range 12-64 months). The female/male ratio was 29/15. Mean age was 50 years (range 22-75 years). The most frequent uveitis etiologies were idiopathic (25 patients, 40.3%) and Behçet’s uveitis. (17 patients, 27.4%) The signedmost common indication for DEX injection was cystoid macular edema together with resistant vitreous haze (26 eyes, 41.9%). Twenty-two eyes (30%) received more than one DEX injection. Mean BCVA was improved from 0.55 logMAR at baseline to 0.38, 0.32, and 0.35 after 1, 3, and 6 months, respectively (p<0.001 for each). Mean CFT was decreased from 386 μm at baseline to 288, 311, and 302 μm after 1, 3, and 6 months, respectively (p<0.001 for each). Mean IOP did not change significantly during follow-up. Five eyes (8%) received topical anti-glaucoma medication (IOP ≥25 mmHg). Eighteen (46%) of 39 phakic eyes underwent cataract surgery during follow-up. Similar efficacy of the DEX implant was observed in eyes that received multiple injections. Systemic immunomodulatory therapy did not change significantly during follow-up.

**Conclusion:**

Intravitreal DEX injection does not alter systemic immunomodulatory therapy, but may facilitate the management of noninfectious uveitis by suppressing local intraocular inflammation. Multiple injections yielded comparable visual and anatomical outcomes to single injections. Follow-up for ocular hypertension and cataract formation are important, especially in eyes receiving multiple injections.

## Introduction

Noninfectious uveitis accounts for 10-15% of all cases of blindness in developed countries.^1^ The most common causes of vision loss are cystoid macular edema (CME), secondary cataract, high intraocular pressure (IOP), and vitreous haze (VH).^2^ The treatment of noninfectious uveitis mainly aims to suppress inflammation and often employs antimetabolites and immunomodulatory agents such as calcineurin inhibitors and biological agents.^3^

Corticosteroids also play an important role in the treatment of uveitis because of their rapid, extensive, and effective anti-inflammatory properties.^4^ However, the use of systemic corticosteroids is limited due to adverse effects such as high blood glucose, systemic hypertension, reduced bone density, depression, and weight gain.^5^ This led to the use of local corticosteroids, which are believed to not cause systemic side effects. However, the periorbital and intravitreal triamcinolone acetonide injections used for this purpose also cause undesirable adverse effects such as cataract and elevated intraocular pressure, and require repeated injections. This in turn led to the introduction of slow-release implants, which are considered safer.^6,7^

Intravitreal dexamethasone implants (Ozurdex, Allergan, Irvine, CA, USA), which are suggested to be safer and have longer lasting effects, were developed for easy injection into the vitreous cavity. The dexamethasone implant (DEX) is a biodegradable polymer composed of a combination of 0.7 mg dexamethasone and poly(lactic-co-glycolic acid).^8^ It slowly dissolves in the vitreous cavity and provides intravitreal dexamethasone release for 6 months. It is indicated for use in cases of CME due to retinal vein occlusions, diabetic macular edema, and noninfectious uveitis.^9,10,11^ The HURON (cHronic Uveitis evaluation of the intRavitreal dexamethasONe implant) trial demonstrated that a single dose injection suppresses inflammation and is effective for up to 6 months in cases of noninfectious uveitis.^11^

The aim of this study was to evaluate the long-term outcomes of intravitreal 0.7 mg dexamethasone implant in eyes with noninfectious uveitis being followed at a single center.

## Materials and Methods

### Patient Selection

This retrospective study included noninfectious uveitis patients over 18 years of age who were treated with DEX injection(s) between July 2015 and December 2017 in the Department of Ophthalmology of Gazi University due to CME and/or refractory VH and intraocular inflammation such as posterior scleritis. All patients had newly started systemic therapy, required no change in existing systemic therapy, or had infrequent acute episodes. The study was approved by the local ethics committee and adhered to the principles of the Declaration of Helsinki. Patients who were not followed up for at least 1 year after injection were not included in the study.

### Data Collection

Patient data analyzed in this study included age, sex, laterality, uveitis diagnosis, indication for DEX implant, anatomical classification of the uveitis, drugs used for systemic therapy before and after injection, number of DEX injections, period between injections if the patient received multiple injections, complications, and total follow-up time. We also evaluated the patients’ best corrected visual acuity (BCVA), intraocular pressure (IOP), anterior segment examination findings (especially lens status), fundus examination findings, central foveal thickness (CFT) measured by optical coherence tomography (OCT), and VH score according to SUN (Standardization of Uveitis Nomenclature Working Group) criteria recorded before and at 1, 3, and 6 months after injection. BCVA values obtained using Snellen chart were converted from decimal system to Logarithm of Minimum Angle of Resolution (logMAR) prior to statistical analysis. CFT measurements made with OCT (Spectralis OCT, Heidelberg Engineering, Heidelberg, Germany) were made using the values automatically acquired by the device.

### Statistical Analysis

SPSS software (version 22.0, SPSS, Inc. Chicago, IL, USA) was used for statistical analysis. Kolmogorov-Smirnov test was used to determine whether the data were normally distributed. For normally distributed variables (first injection BCVA, CFT, and IOP), paired t-test was used to evaluate changes in BCVA, CFT, and IOP values between baseline and the other time points. For variables that did not show normal distribution (second and third injection BCVA, CFT, and IOP), these comparisons were made using Wilcoxon signed-rank test. Changes with p values <0.05 were considered significant.

## Results

Sixty-two eyes of 44 patients were included in the study. The patients’ demographic characteristics, uveitis diagnoses and anatomical locations, and systemic therapies received are shown in Table 1. The most common etiology of noninfectious uveitis was idiopathic (40.3%), followed by Behçet’s disease (27.4%). Two patients (3.2%) who had received antituberculous therapy for ocular tuberculosis but subsequently developed a Jarish–Herxheimer-like inflammatory reaction were also included in the noninfectious uveitis group in this study. The most common anatomic involvement was posterior uveitis (53.2%). In terms of treatment, 40.9% of the patients were not receiving systemic therapy, while 17 patients were receiving systemic corticosteroids at a median dose of 16 mg (range: 2-72 mg). Indications for intravitreal DEX injection are shown in Table 2. The most common indication for DEX was CME (44 eyes, 70.9%). Twenty-six eyes (41.9%) had both CME and refractory VH. The clinical characteristics of the patients included in the study are shown in Table 3. The mean initial BCVA was 0.55±0.46. VH score was 2+ or higher in 24 eyes (39%). Twenty-three eyes (37.1%) had prior cataract surgery, while 25 (40%) eyes were phakic with clear lens. Twenty-two (35.4%) of the 62 eyes received multiple DEX injections.

**Figure 1:**
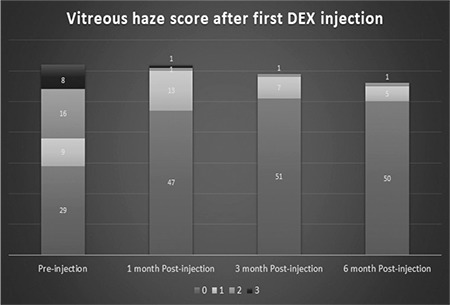
Distribution of the patients’ vitreous haze scores before and at 1, 3, and 6 months after the first intravitreal dexamethasone implant injection. Vitreous haze decreased markedly in the first 3 months and this effect persisted to 6 months DEX: Dexamethasone

Clinical outcomes after intravitreal DEX injection are shown in Tables 4, 5, and 6. BCVA was significantly increased at 1, 3, and 6 months after the first DEX injection compared to baseline (p<0.001). Although IOP was significantly higher than baseline at 1 month after injection (p=0.007), it did not differ significantly at 3 or 6 months (p=0.202 and 0.848, respectively). According to CFT measurements, CME decreased significantly compared to baseline values at 1, 3, and 6 months after treatment (p=0.001, 0.002, 0.004, respectively). VH was detected in 33 (53%) eyes before injection and 6 (10%) eyes 6 months after injection (Figure 1). Reductions in VH from baseline examination results were significant at 1, 3, and 6 months (p<0.001).

**Figure 2:**
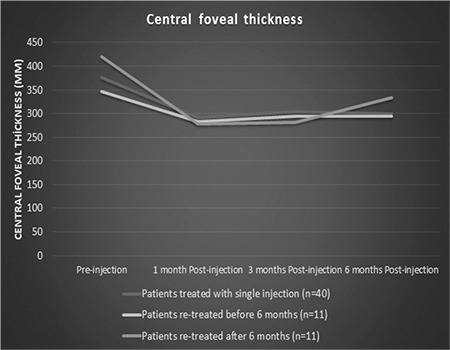
Central foveal thickness measurements before and 6 months after the first intravitreal dexamethasone implant injection in patients who received a single dose and those who received repeated doses after intervals of at least 6 months. The change in central foveal thickness was similar in all groups

In eyes treated with a second DEX injection (n=22, 35%), the median interval between the injections was 4.5 months (range: 3-25 months). Only 3 eyes (4%) received a third DEX injection. Eleven eyes (17%) required repeat DEX injection within 6 months. Compared to eyes that received a single dose of DEX and those who received repeat DEX after an interval of 6 months or longer, these eyes showed similar improvement in BCVA and reduction in CFT, but IOP increased during the first months (Figure 2). Changes in BCVA, CFT, and IOP according to number of DEX injections are shown in Tables 4, 5, and 6, respectively. Eyes that received a second DEX injection showed significant increases in BCVA and decreases in CFT at 1, 3, and 6 months compared to baseline values, similar to after the first injection. IOP did not change significantly from baseline at any of the time points. In eyes that received a third DEX injection, BCVA, CFT, and IOP values did not show significantly changes at 1, 3, or 6 months after injection when compared with baseline values. Eyes that received a single injection and those that received two injections had statistically equivalent BCVA, CFT, and IOP values at baseline and all post-injection time points.

At the beginning of follow-up, 25 of the 62 eyes were phakic with clear lens, 23 were pseudophakic, and 14 were phakic with cataract. At final examination, 9 of the 62 eyes were phakic with clear lens, 41 were pseudophakic, and 12 were phakic with cataract. Of the 18 eyes that were phakic at the beginning of follow-up and underwent cataract surgery during the follow-up period, 10 received a single DEX injection and 8 received two doses. Of the eyes that were initially phakic with clear lenses and developed cataract during follow-up but did not undergo surgery, 4 eyes received a single dose of DEX, 1 eye received two doses, and 1 eye received three doses. Five patients required topical antiglaucoma treatment during follow-up (IOP >20 mmHg). None of the patients required surgery due to high IOP. Prior to the first DEX injection, 28 (63.6%) of the 44 patients were receiving systemic therapy, with 16 (36.4%) using systemic steroids either alone or in combination with other drugs. At final examination, a total of 25 patients (56.9%) were receiving systemic therapy, with 8 (18.2%) patients receiving systemic steroid therapy either alone or in combination with other drugs (Table 7). There was no significant change when compared with their initial systemic therapies.

## Discussion

In this study, we investigated the effectiveness of intravitreal DEX injections in noninfectious uveitis based on real-life outcomes. The results of this single-center, retrospective study showed that DEX injection was beneficial in suppressing ocular inflammation and that similar results could be obtained with repeated injections, but patients should be monitored closely for cataract and IOP. In addition, DEX injection was shown to facilitate systemic disease control and reduce the use of systemic steroids, but did not have a significant effect on systemic immunosuppressive therapy.

Suppressing intraocular inflammation and preserving vision are the main goals in the treatment of noninfectious uveitis. It was previously reported in the HURON trial that BCVA increases and is maintained for at least 6 months after DEX injection.^11^ Although the HURON trial demonstrated the utility of DEX in the treatment of noninfectious uveitis, it was conducted in a limited patient group and provided short-term results, and thus provides limited information regarding patients encountered in real practice. In 2014, Zarranz-Ventura et al.^12^ published a multicenter retrospective cohort study of DEX results in 82 eyes of 63 patients diagnosed with noninfectious uveitis. They reported statistically significant improvements in BCVA, CFT, and VH, though during the 1-year follow-up period, 40.7% of the patients required a second injection at a mean of 6.6 months. Tomkins-Netzer et al.^13^ reported in another retrospective study that DEX remained effective for a median of 6 months. In their prospective study, Pohlmann et al.^2^ showed that vision improved from 1 month and was preserved until 6 months. In the present study, visual acuity was significantly increased at 1, 3, and 6 months of follow-up compared to baseline BCVA and was well preserved. In this study, 31% (n=22) of the 62 eyes required a second dose injection at a median of 4.7 months, and 3 eyes (5%) received three doses of DEX.

The most common cause of vision loss in cases of noninfectious uveitis is CME.^14,15^ Reduction in the frequency of CME results in improved visual acuity. Pohlmann et al.^2^ determined that the effect of DEX on CME varies depending on the etiology. They reported that the decrease in CME lasts longer in patients with idiopathic uveitis than in cases of uveitis associated with sarcoidosis or other systemic diseases, and that CME decreases more rapidly in patients with birdshot retinochoroidopathy. It has also been reported that response to DEX is unaltered in chronic CME, and that visual improvement was achieved upon the complete resolution of CME even in cases resistant to other therapies.^16,17^ The frequency of re-injection is higher in patients with chronic CME.^12,16^ Our shorter re-injection period may be associated with the nonrandom patient selection, due to the probably long-term intraocular inflammation having limited response to the injection, the presence of chronic CME, or insufficiently suppressed systemic disease.

VH regresses as intraocular inflammation is suppressed. DEX suppresses local inflammation effectively as long as it remains in the vitreous.^2,12,18^ Reduction in VH also increases visual acuity. In the present study, 33 of the 62 eyes had VH scores of 1+ or higher before the first injection, while only 6 eyes had VH scores of 1+ or higher 6 months after injection (1+ in 5 eyes, 2+ in 1 eye). DEX injection decreases VH in the long term by locally suppressing intraocular inflammation.

Management of noninfectious uveitis is challenging due to the severe and frequent side effects of systemic steroids, the short-lasting effect of off-label periocular or intravitreal triamcinolone injections, and IOP elevation frequently caused by these injections.^11^ DEX has emerged as a safe and long-acting treatment for local inflammation control in combination with immunomodulatory and immunosuppressive systemic therapies.^11^ With efficacy in noninfectious uveitis demonstrated by the HURON trial, DEX has provided intraocular inflammation control for approximately 6 months as well as significant increases in BCVA and significant decreases in VH and CFT. IOP increased by less than 10%. In a retrospective study of 1110 eyes treated with DEX, it was reported that only 65 eyes required topical antiglaucoma medication, 5 patients underwent selective laser trabeculoplasty, and none of the patients required surgery.^19^ Similarly, in the present study we observed statistically significant increase in BCVA and decrease in CFT and VH. In addition, IOP elevation requiring antiglaucoma medication (>25 mmHg) occurred in 5 of the 62 eyes in our study, consistent with the results of the HURON trial.

The main objective of DEX injection is local inflammation control. The main treatment approach for noninfectious uveitis is to control inflammation with systemic immunosuppressive agents and reduce the frequency of acute attacks. DEX injections facilitate rapid inflammation control in patients who do not have frequent exacerbations or have recently started receiving systemic therapy. In addition, it enables the rapid regression of pathologies that reduce vision, such as VH and CME. For patients already receiving systemic immunosuppressive therapy, DEX injection helps achieve local inflammation control before deciding to change their treatment regimen, which allows patients to continue with the same treatment they are used to and do not experience side effects with. Although the number of patients using systemic steroids decreased after DEX injection in our study, the number of patients receiving immunosuppressive therapy remained unchanged. In the earlier Multicenter Uveitis Steroid Treatment (MUST) trial of the fluocinolone acetonide implant, it was reported that it reduced the need for systemic immunosuppressive therapy and that disease control could be achieved with intravitreal injection.^20^ Tomkins-Netzer et al.^13^ found that 21 of the 33 eyes in their study did not require immunosuppressive therapy after a single DEX injection. In contrast, Tsang et al.^17^ found that patients not receiving systemic therapy showed poorer response to DEX injection. Fabiani et al.^21^ reported that the steroid dose given to patients was significantly reduced after DEX injection and described intravitreal DEX injection as a systemic steroid-sparing treatment. Although intravitreal DEX injection seems to reduce the need for systemic steroids, in general there is no evidence demonstrating its effect on systemic immunosuppressive therapy. Well-designed prospective studies on this subject are needed.

### Study Limitations

One of the limitations of the HURON trial is that the patients were followed up for only 6 months and no long-term results are presented. Therefore, it does not provide sufficient information about the development of cataract in the longer term. In the MUST trial of fluocinolone acetonide implant, the prevalence of cataract was 80%.^20^ Much lower cataract rates have been reported after DEX injection in other studies.^12,13,16^ Nobre-Cardoso et al.^22^ reported that all patients in their study who developed cataract had received multiple injections. In their prospective, single-center study, Pohlmann et al.^2^ showed that the rate of pseudophakia was 50% in patients who were followed for an average of 22 months and increased to 94% before the fourth injection. In the present study, 23 of the 62 eyes were pseudophakic initially and 41 eyes were pseudophakic at the end of the mean 20-month follow-up period. Patients injected with DEX should be carefully monitored for cataract development in the long term, especially if repeated injections are needed.

The limitations of our study stem from its retrospective nature and small patient sample. Despite their small numbers, however, the inclusion of patient groups with various intraocular inflammation etiologies is a better representation of the patient profile encountered in real practice, which is a strength of our study.

## Conclusion

In conclusion, intravitreal DEX injection is useful for suppressing intraocular inflammation, provides good visual and anatomical results in the long term, and preserves these effects with repeated injections. However, although it may seem safer than other intravitreal steroid treatments in terms of IOP and cataract formation, patients still require close follow-up. DEX appears to reduce the need for systemic steroids, but this phenomenon and its effect on systemic immunosuppressive therapies must be clarified by long-term prospective studies.

## Figures and Tables

**Table 1:**
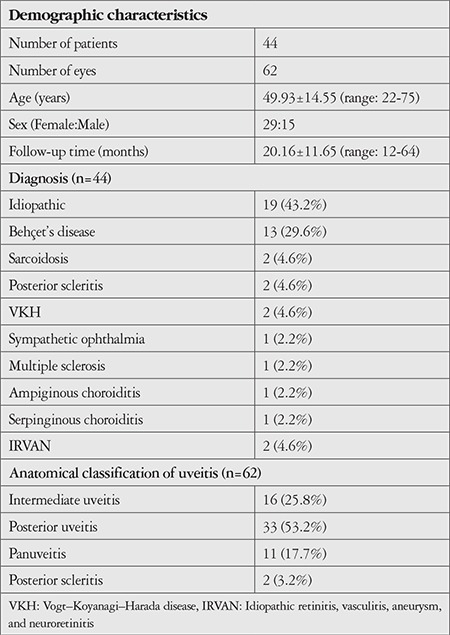
Demographic characteristics and uveitis diagnoses, locations, and systemic treatments in the study patients

**Table 2:**
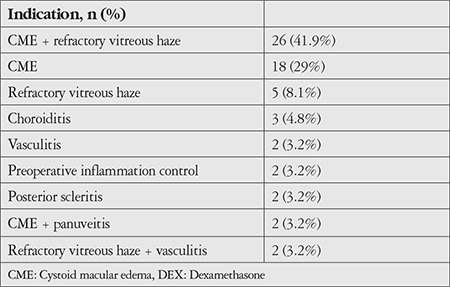
Indications for intravitreal DEX implantation (n=62)

**Table 3:**
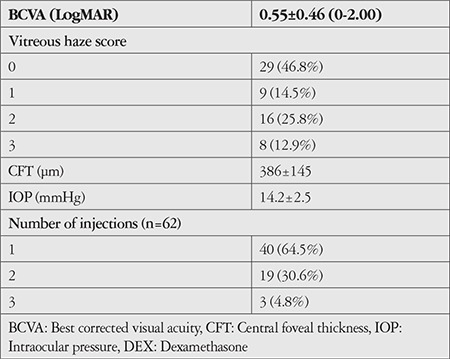
Initial clinical characteristics of eyes treated with intravitreal DEX (n=62)

**Table 4:**
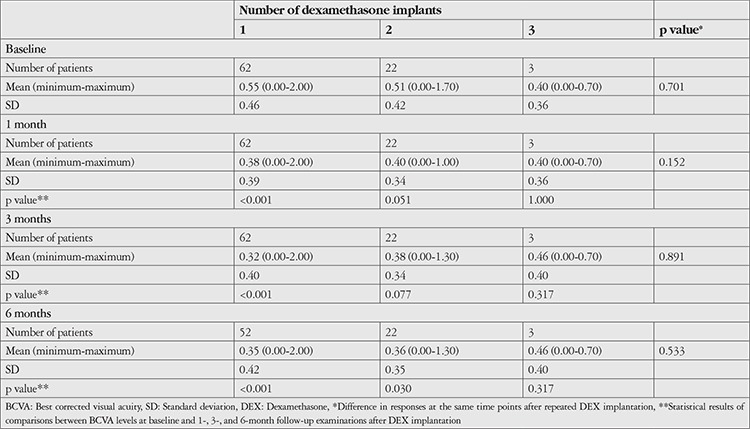
BCVA levels after intravitreal DEX implantation

**Table 5:**
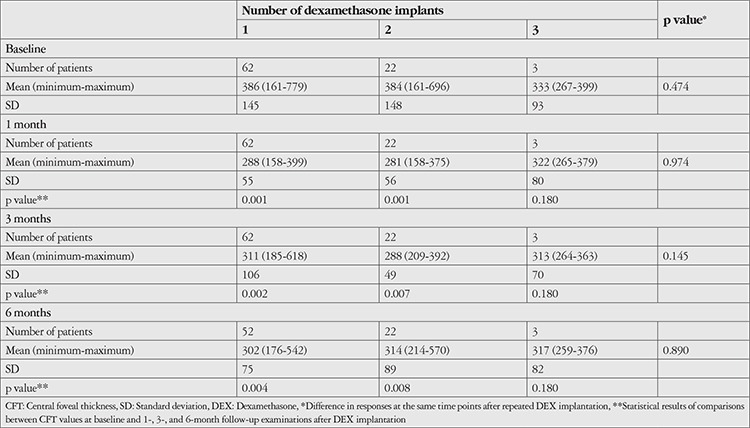
Central foveal thickness measurements (μm) after intravitreal DEX implantation

**Table 6:**
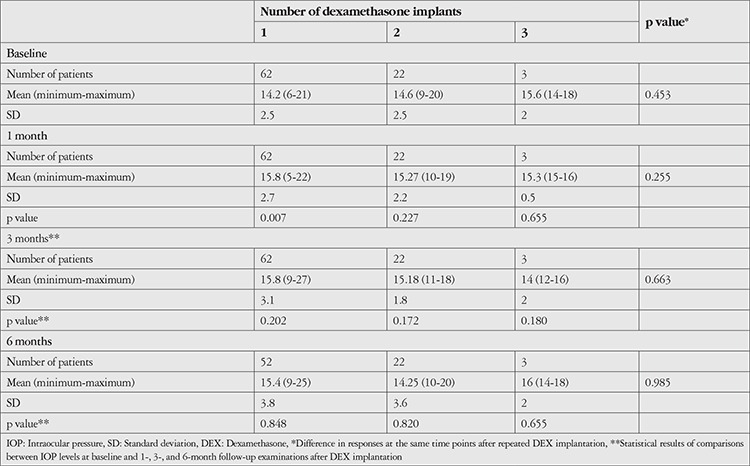
Intraocular pressure measurements (mmHg) after intravitreal DEX implantation

**Table 7:**
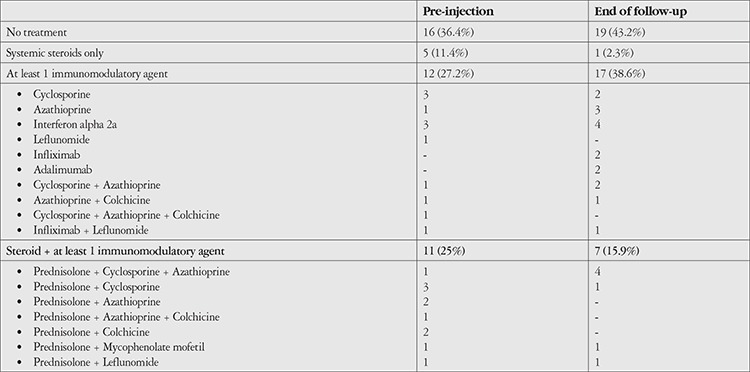
Number of patients using systemic drugs at initial and final examination (n=44)
